# Systemic Immune-Inflammation Index Is a Prognostic Factor for Breast Cancer Patients After Curative Resection

**DOI:** 10.3389/fonc.2021.570208

**Published:** 2021-12-01

**Authors:** Wen Li, Guangzhi Ma, Yunfu Deng, Wenjie Chen, Zhenkun Liu, Fang Chen, Qiang Wu

**Affiliations:** ^1^ Lung Cancer Center & Institute, West China Hospital, Sichuan University, Chengdu, China; ^2^ Cancer Center, West China Hospital, Sichuan University, Chengdu, China; ^3^ Department of Thoracic Surgery, The Third Affifiliated Hospital of Kunming Medical University (Yunnan Cancer Hospital, Yunnan Cancer Center), Kunming, China; ^4^ Breast Center, West China Hospital, Sichuan University, Chengdu, China

**Keywords:** breast cancer, SII, inflammation, prognostic factor, survival

## Abstract

**Background:**

The preoperative systemic immune-inflammation index (SII) is correlated with prognosis in several malignancies. The aim of this study was to investigate the prognosis value of SII in patients with resected breast cancer.

**Materials and Methods:**

A total of 784 breast cancer patients who underwent surgical resection were consecutively investigated. The optimal cutoff value of SII was evaluated using the receiver operating characteristic (ROC) curve. The collection of SII with clinicopathological characteristic and prognosis was further evaluated.

**Results:**

The optimal cutoff value for SII in the prediction of survival was 514 according to ROC curve analysis. A high SII was significantly correlated with younger age (*P* = 0.037), PR status (*P* < 0.001), and HER2 status (*P* = 0.035). Univariate analysis revealed that SII (*P* < 0.001), T-stage (*P* < 0.001), lymph node involvement post-surgery (*P* = 0.024), and histological grade (*P* < 0.001) were significantly related to DFS, and SII (*P* < 0.001), T-stage (*P* = 0.003), lymph node involvement post-surgery (*P* = 0.006), and histological grade (*P* < 0.001) were significantly associated with OS. In multivariate analysis, a high SII was an independent worse prognostic factor for DFS (HR, 4.530; 95% CI, 3.279-6.258; *P* < 0.001) and OS (HR, 3.825; 95% CI, 2.594-5.640; *P* < 0.001) in all the enrolled patients. Furthermore, subgroup analysis of molecular subtype revealed that SII was significantly associated with prognosis in all subtypes.

**Conclusion:**

Preoperative SII is a simple and useful prognostic factor for predicting long-term outcomes for breast cancer patients undergoing surgery.

## Introduction

Breast cancer is one of the most commonly diagnosed malignancies and the leading cause of cancer death in women worldwide (1). Despite developments in radiotherapy, chemotherapy, and targeted therapy, surgery is still the main treatment method for localized breast cancer patients. However, its clinical outcome remains unsatisfactory because an appreciable patient ultimately develops local recurrences or distant metastases after resection (2). Therefore, identifying reliable potential biomarkers for stratifying patients who are likely to have a high risk of recurrence or mortality is crucial to the selection of appropriate treatment strategies (3).

Tumor-promoting inflammation and immune system role in cancer surveillance and elimination are enabling hallmarks for malignant cells (4, 5). Systemic inflammatory responses can influence cancer formation and progression at the molecular level, such as DNA damage and cell proliferation (6). Except for tumor cells, immune and inflammatory cells, including neutrophils, platelets, and lymphocytes, contribute to malignant cell invasion in the peripheral blood; hence, tumor cells can survive and reseed in distant organs (7). Several inflammation and immunity-based indicators, including lymphocyte count, neutrophil-lymphocyte ratio (NLR), and platelet-lymphocyte ratio (PLR), have been used in predicting survival outcomes (8–11). Systemic immune-inflammation index (SII), calculated by lymphocyte, neutrophil, and platelet counts, reflects the balance of host inflammatory and immune status and is an established prognostic factor in several malignies (12–14). However, the prognostic value of SII in breast cancer patients remains unclear. In the present study, we aimed to evaluate the prognostic value of SII in patients after curative resection for breast cancer.

## Patients and Methods

### Patients and Follow-Up

We retrospectively identified consecutive breast cancer patients who underwent surgery at West China Hospital of Sichuan University from June 2012 to July 2015. Inclusion criteria were as follows: (1) patients received surgery; (2) histologically confirmed breast cancer; and (3) patients with sufficient clinicopathological date and clinical information. Exclusion criteria were (1) ductal carcinoma in situ; (2) patients with metastatic disease before surgery; (3) patients with infections, inflammatory, hematologic, or autoimmune diseases; (4) patients received neoadjuvant chemotherapy before surgery; and (5) male breast cancer patients. Histopathological data and clinical information were obtained.

All the patients were followed up every three months in the first three years after operation, every six months in the next five years, and once a year thereafter. Disease free survival (DFS) was defined as the time from the date of diagnosis to the date of disease relapse/the last follow-up date. Overall survival (OS) was defined as the interval period from the first diagnosis to the death/final follow-up. The present study was approved by the Research Ethics Committee of West China Hospital of Sichuan University, and a written informed consent was obtained from each participant in accordance with the policies of the committee.

### Pathology Methods and Molecular Subtypes

Estrogen receptor (ER), progesterone receptor (PR), and human epidermal growth factor receptor 2 (HER2) statuses and Ki67 expression were determined by immunohistochemical staining. The cutoff value for positive ER or PR was ≥1% of stained cell, and the cutoff value of high Ki-67 was ≥14% of immunoreactive tumor cell nuclei. Additionally, a value of 0 or 1+ was reported as HER2 negative, and 3+ was considered HER2 positive. Fluorescence *in situ* hybridization was performed when the level of staining was 2+.

For the molecular subtypes, all the patients were classified as luminal A (ER+ and/or PR+, HER2-, Ki-67 <14), luminal B (ER+ and/or PR+, Ki-67 ≥14% or HER2+/any Ki-67), HER2-enriched (ER-, PR-, HER2+, any Ki-67), or triple-negative (ER-, PR-, HER2-, any Ki-67) breast cancer (TNBC), according to the St. Gallen Expert Consensus in 2013 (15).

### Data Collection and Definitions

A complete preoperative blood cell count was obtained within seven days before surgery. According to previous studies, SII was calculated using the formula SII = P × N/L, where P, N, and L represent the absolutely platelet count (10^9^/L), neutrophil count (10^9^/L), and lymphocyte count (10^9^/L), respectively (16).

### Determination of the SII Cutoff Value

We used the receiver operating characteristic (ROC) curve to determine the sensitivity and specificity of SII for 5-year survival, and the Youden index was used in calculating and selecting the optimal cutoff value of SII.

### Statistical Analysis

The associations between SII and clinicopathologic characteristics were analyzed using *X^2^*-test. The survival curves of DFS and OS were depicted using the Kaplan–Meier method and analyzed using the log-rank test. Univariate and multivariate analyses were performed using the Cox proportional hazards model, and the hazard ratios (HRs) and corresponding 95% confidence intervals (CIs) of each factor were reported. Statistical analyses were performed using the SPSS (version 23.0) software package (SPSS Inc., Chicago, IL, USA). A two-tailed *P* value of <0.05 was considered statistically significant.

## Results

### Characteristics of Patients

**Figure 1:**
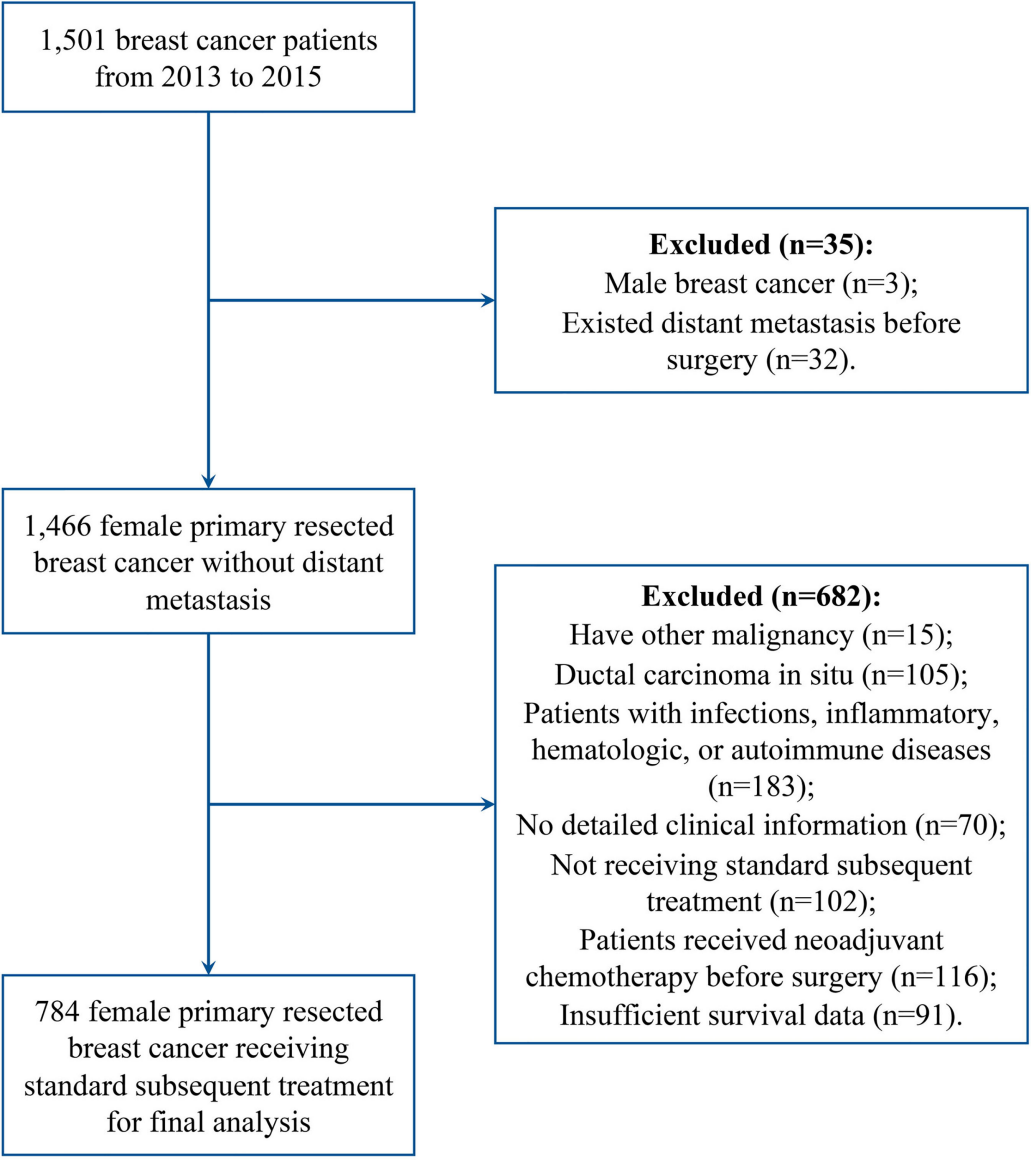
Flow chart of patient selection.

There were 1501 breast cancer patients who were diagnosed with breast cancer in West China Hospital of Sichuan University from June 2012 to July 2015. Among all the 1501 breast patients, 717 were excluded as they not meet the inclusion criteria of the study, and then a total of 784 female breast cancer patients who underwent surgery were included in this study (**Figure 1**). The median age was 49 years (20-89 years), and the median follow-up period was 65.5 months (3-91.5months). Tumor relapse occurred in 157 patients, 108 of which died. A total of 170 (21.7%) patients were younger than 40 years old, and 450 (57.4%) patients exhibited positive PR expression. The majority of the patients had positive ER expression (62.6%), high Ki67 proliferation (63.1%), and negative HER2 expression (75.0%). The clinical and pathologic characteristics of all the included patients are shown in **Table 1**.

**Table 1:** Association of the systemic immune-inflammation index (SII) with the patient and tumor characteristics.

	Total	Low SII	High SII	*P* value
**Age**	784	562	222	0.037
≤40	170 (21.7%)	111 (19.8%)	59 (26.6%)	
>40	614 (78.3%)	451 (80.2%)	163 (73.4%)	
**ER**				0.416
−	293 (37.4%)	215 (38.3%)	78 (35.1%)	
+	491 (62.6%)	347 (61.7%)	144 (64.9%)	
**PR**				< 0.001
−	334 (42.6%)	273 (48.6%)	61 (27.5%)	
+	450 (57.4%)	289 (51.4%)	161 (72.5%)	
**HER2**				0.035
−	588 (75.0%)	433 (77.0%)	155 (69.8%)	
+	196 (25.0%)	129 (23.0%)	67 (30.2%)	
**Ki-67 status**				0.396
−	289 (36.9%)	202 (35.9%)	87 (39.2%)	
+	495 (63.1%)	360 (64.1%)	135 (60.8%)	
**pT Stage**				0.949
1	249 (31.8%)	178 (31.7%)	71 (32.0%)	
2	450 (57.4%)	321 (57.1%)	129 (58.1%)	
3	60 (7.7%)	45 (8.0%)	15 (6.8%)	
4	25 (3.2%)	18 (3.2%)	7 (3.2%)	
**pN Stage**				0.377
0	381 (48.6%)	283 (50.4%)	98 (44.1%)	
1	276 (35.2%)	188 (33.5%)	88 (39.6%)	
2	100 (12.8%)	71 (12.6%)	29 (13.1%)	
3	27 (3.4%)	20 (3.6%)	7 (3.2%)	
**Histological grade**				0.337
I-II	549 (70.0%)	388 (69.0%)	161 (72.5%)	
III	235 (30.0%)	174 (31.0%)	61 (27.5%)	
**Surgery type**				0.538
Mastectomy	621 (79.2%)	442 (78.6%)	179 (80.6%)	
BCS	163 (20.8%)	120 (21.4%)	43 (19.4%)	
**Chemotherapy**				0.369
Yes	281 (35.8%)	196 (34.9%)	85 (38.3%)	
No	503 (64.2%)	366 (65.1%)	137 (61.7%)	
**Radiotherapy**				0.627
Yes	285 (36.4%)	205 (36.5%)	80 (36.0%)	
No	499 (63.6%)	357 (63.5%)	142 (64.0%)	
**Hormonal therapy**				0.054
Yes	510 (65.1%)	354 (63.0%)	156 (70.3%)	
No	274 (34.9%)	208 (37.0%)	66 (29.7%)	
**Target therapy**				0.220
Yes	156 (19.9%)	118 (21.0%)	38 (17.1%)	
No	628 (80.1%)	444 (79.0%)	184 (82.9%)	

PR, progesterone receptor; ER, estrogen receptor; TNBC, triple-negative breast cancer; BCS, breast conserving surgery.

### ROC Analysis for the Prediction of Survival

**Figure 2:**
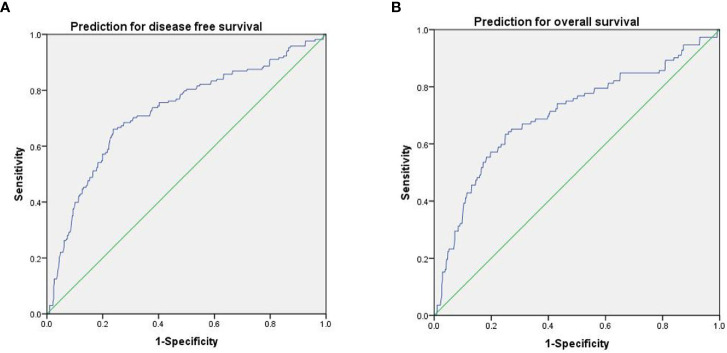
ROC curves of SII for predicting DFS **(A)** and OS **(B)**.

The results of ROC analysis showed that the area under the ROC curve (AUC) of SII for predicting DFS and OS were 0.724 (*P* < 0.001; 95% CI, 0.679-0.770) and 0.703 (*P* < 0.001; 95% CI, 0.645-0.761), respectively (**Figures 2A, B**). According to the Youden index, an SII value of 514 was the optimal cutoff value, and sensitivity and specificity were 63.4% and 75.0% for OS, respectively. The patients were then stratified into two groups, and 562 (71.7%) patients showed low SII, whereas 222 (28.3%) patients had high SII.

### Relationships Between SII and Clinicopathological Characteristics

As shown in **Table 1**, SII was significantly related to younger age (*P* = 0.037), positive PR expression (*P* < 0.001), and positive HER2 expression (*P* < 0.001) but not to Ki67 expression level, ER status, T-stage, N-stage, high histological grade, surgery type, chemotherapy, hormonal therapy, target therapy and radiotherapy.

### Correlations of the SII With Survival

**Figure 3:**
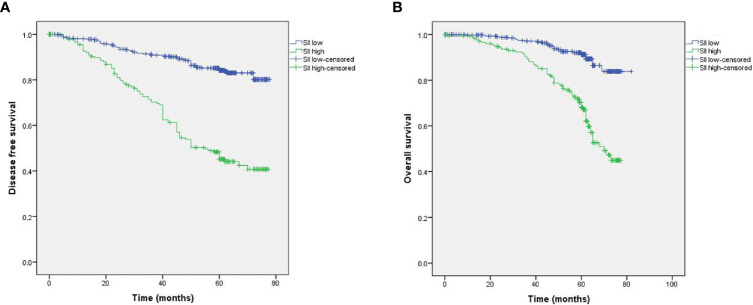
Kaplan-Meier survival analyses of the correlation between SII and survival among breast cancer patients: DFS **(A)** and OS **(B)**.

The Kaplan-Meier survival curves analysis showed that high SII is a poor prognostic factor for DFS and OS. The 5-year survival rates for DFS and OS in the entire cohort were 79.9% and 86.2%, respectively. The 5-year DFS rate in the high SII group was significantly shorter than in the low SII group (57.0% and 84.1%, respectively, *P* < 0.001; HR, 4.296; 95% CI, 2.906-6.350; **Figure 3A**). Moreover, the corresponding 5-year OS rate for patients in the high SII group (72.4%) was significantly shorter than those in the low SII group (91.2%; *P* < 0.001; HR, 4.304; 95% CI, 3.125-5.929; **Figure 3B**).

Univariate Cox regression analysis revealed that SII (*P* < 0.001), T-stage (*P* < 0.001), lymph node involvement post-surgery (*P* = 0.024), and histological grade (*P* < 0.001) were significantly related to DFS, and SII (*P* < 0.001), T-stage (*P* = 0.003), lymph node involvement post-surgery (*P* = 0.006), and histological grade (*P* < 0.001) were significantly associated with OS (**Tables 2**, **3**).

**Table 2:** Results of the analysis of the prognostic factors for disease free survival.

	Univariate analysis		Multivariate analysis	
	HR (95% CI)	*P* value	HR (95% CI)	*P* value
**SII**		<0.001		<0.001
Low	1.00		1.00	
High	4.304 (3.125 -5.929)		4.530 (3.279 -6.258)	
**Patient age**		0.250		
≤40	1.00			
>40	0.807 (0.560 -1.163)			
**ER**		0.824		
–	1.00			
+	0.964 (0.699 -1.330)			
**PR**		0.732		
–	1.00			
+	0.947 (0.692 -1.295)			
**HER2**		0.363		
–	1.00			
+	0.820 (0.535 -1.257)			
**Ki-67 status**		0.058		
–	1.00			
+	1.596 (0.983 -2.590)			
**pT Stage**		<0.001		<0.001
1	1.00		1.00	
2-4	1.340 (1.163 -1.543)		1.368 (1.192 -1.571)	
**pN Stage**		0.024		0.027
–	1.00		1.00	
+	1.182 (1.022 -1.368)		1.179 (1.019 -1.364)	
**Histological grade**		<0.001		0.034
I-II	1.00		1.00	
III	1.795 (1.277-2.521)		1.595 (1.036 -2.451)	
**Surgery type**		0.344		
Mastectomy	1.00			
BCS	1.207 (0.821-1.769)			
**Chemotherapy**		0.08		
No	1.00			
Yes	1.340 (0.968-1.852)			
**Radiotherapy**		0.121		
No	1.00			
Yes	0.775 (0.561-1.068)			
**Hormonal therapy**		0.110		
No	1.00			
Yes	0.767 (0.553-1.060)			
**Target therapy**		0.253		
No	1.00			
Yes	1.259 (0.851-1.858)			

**Table 3:** Results of the analysis of the prognostic factors for overall survival.

	Univariate analysis		Multivariate analysis	
	HR (95% CI)	*P* value	HR (95% CI)	*P* value
**SII**		<0.001		<0.001
Low	1.00		1.00	
High	4.296 (2.906 -6.350)		3.825 (2.594 -5.640)	
**Patient age**		0.745		
≤ 40	1.00			
>40	0.927 (0.589 -1.460)			
**ER**		0.187		
–	1.00			
+	0.774 (0.528 -1.133)			
**PR**		0.517		
–	1.00			
+	0.883 (0.605 -1.288)			
**HER2**		0.733		
–	1.00			
+	0.916 (0.552 -1.519)			
**Ki-67 status**		0.112		
–	1.00			
+	1.636 (0.892 -3.000)			
**pT Stage**		0.003		<0.001
1	1.00		1.00	
2-4	1.300 (1.093 -1.546)		1.377 (1.162 -1.632)	
**pN Stage**		0.006		0.035
–	1.00		1.00	
+	1.272 (1.070 -1.512)		1.211 (1.014 -1.447)	
**Histological grade**		<0.001		0.006
I-II	1.00		1.00	
III	2.058 (1.364-3.101)		1.937 (1.207 -3.106)	
**Surgery type**		0.569		
Mastectomy	1.00			
BCS	1.147 (0.721-1.820)			
**Chemotherapy**		0.151		
Yes	1.00			
No	0.752 (0.508-1.109)			
**Radiotherapy**		0.533		
Yes	1.00			
No	0.885 (0.598-1.305)			
**Hormonal therapy**		0.212		
No	1.00			
Yes	0.779 (0.525-1.153)			
**Target therapy**		0.405		
No	1.00			
Yes	1.223 (0.764-1.959)			

Multivariate analysis showed that SII, T-stage, lymph node involvement post-surgery, and histological grade had significant associations with DFS and OS (**Tables 2**, **3**).

**Figure 4:**
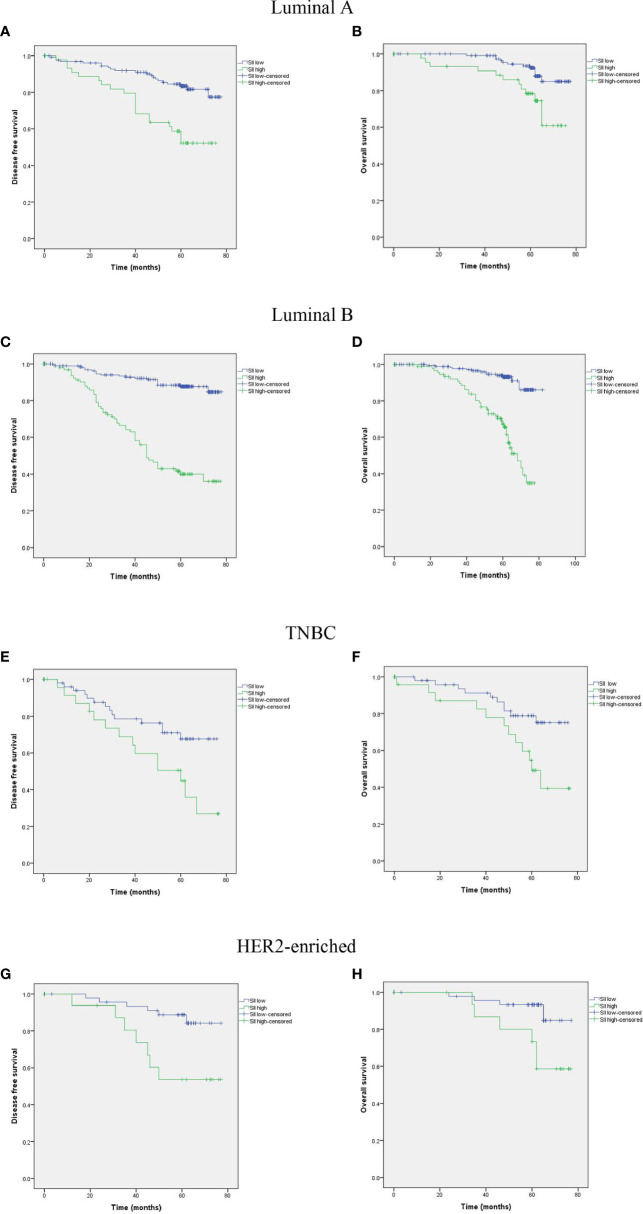
Kaplan-MeierKaplan-Meier survival analyses of DFS and OS according to SII among patients in Luminal A **(A, B)**, Luminal B **(C, D)**, TNBC **(E, F)**, and HER2-enriched **(G, H)** subgroups.

Subgroup analysis by subtype of breast cancer was performed. Among the 784 enrolled breast cancer patients, 235 (30.0%) were classified as luminal A subtype, 368 (46.9%) were luminal B subtype, 108 (13.8%) were HER2 subtype, and 73 (9.3%) were TNBC subtype. The results showed that a high SII was significantly associated with poor prognosis in all the four subtypes (**Figure 4**).

## Discussion

In recent years, inflammation has been demonstrated to be a vital factor in tumor growth, invasion, and metastasis, and the association of inflammation with several malignancies has been revealed (17–20). Many studies have investigated SII in various cancers, including esophageal squamous cell carcinoma, non-small cell lung cancer, hepatocellular carcinoma, and germ-cell tumors (7, 21–23). However, reports on the prognostic significance of SII in breast cancer are rare. In the present study, we assessed the prognostic value of SII in breast cancer patients, and the results showed that SII score obtained before surgery is an independent prognosis factor for breast cancer patients, and a high SII is associated with poor DFS and OS.

Platelets play vital roles not only in classical hemostatic function but also in creating a hypercoagulable environment that can mediate tumor progression. Specifically, platelets induce tumor growth and promote tumor-associated vasculature development and tumor invasion and metastasis (24). Cancer cells can interact with platelets through cell receptors and signaling molecules, such as the ADP, glycoproteins, P-selectin, and thrombin, and platelets can bind to tumor cells and directly induce tumor growth and metastasis by releasing pro-tumor angiogenic and growth factors once the platelets are activated (25). In the metastatic process of tumor cells, platelets can protect tumor cells from high-velocity forces and immunosurveillance, allowing the establishment of a premetastatic niche. Moreover, platelets can also protect circulating tumor cells from shear stress during cell circulation (6, 26). Tumor cells can activate platelets, which in turn secrete transforming growth factor β and platelet-derived growth factor and induce tumor cell epithelial–mesenchymal transition (EMT) (27, 28). Platelets also increase tumor cell metastatic potential by activating the TGFb-1 and NF-kB pathways, which are responsible for the EMT (29). A high platelet count is associated with increased metastasis and poor outcomes in multiple cancers (30, 31).

The immune response of a host to a malignancy is lymphocyte dependent and plays a vital role in tumor defense by inhibiting tumor cell proliferation, invasion, and migration (32). Meanwhile, lymphocytes can release cytokines (such as IFN-β and TNF-α) that are associated with improved prognosis in several cancers (33). By contrast, neutrophils facilitate tumor cell adhesion and the seeding of distant organs by secreting growth factors, including VEGF and proteases (34–38). Some *in vitro* trials revealed that the cytolytic activities of lymphocytes decrease when co-cultured with neutrophils (39). Calculated from these two parameters, NLR can result in better prediction results than individual factors, and numerous studies demonstrated the prognostic value of NLR in cancer patients (40–42). Robinson et al. in their multicenter cohort study revealed that NLR is associated with the volume of melanoma at presentation and may predict occult sentinel lymph metastases (43). In addition, a meta-analysis showed that a raised baseline NLR is related to nearly twice the risk of recurrence in melanoma (44).

Given the significance of platelets, neutrophils, and lymphocytes in prognosis prediction in cancer patients, an elevated preoperative SII usually indicates elevated inflammatory status and weak immune response. Li et al. found that preoperative SII is a prognostic indicator for intrahepatic cholangiocarcinoma and patients with increased SII level are associated with poor OS and early tumor recurrence (45). Wang et al. found the role of SII in non-small cell lung cancer patients in a meta-analysis (46). Preoperative elevated SII can be an independent prognostic factor for bladder cancer patients who underwent radical cystectomy (47). Besides, a relevant study also indicated that the DFS and OS time in patients with low SII would have survival longer than those patients with high SII in patients with breast cancer undergoing neoadjuvant chemotherapy, which is consistent with our results (48). Moreover, a meta-analysis involving 2642 patients suggest that an elevated SII predicts poor survival outcomes and is associated with clinicopathological features that indicate tumor progression of breast cancer (49).

SII is calculated based on standard laboratory tests on total platelet, neutrophil, and lymphocyte counts, and all these parameters are routinely measured in the clinical setting. Thus, SII might be a potential marker for tumor recurrence surveillance in breast cancer patients undergoing potentially curative resection; moreover, we hope that the SII may be used in combination with other biomarkers and serve a useful index for evaluating the risk of breast cancer to identify subgroups of patients with poor prognosis and offer therapeutic strategies for breast cancer patients.

This study has several limitations. Firstly, our study is a retrospective study and all the samples were enrolled at a single center. Secondly, with the subgroup analysis, the numbers of patients are less and may influence the outcomes. Thirdly, although SII is an independent predictor, the SII is a nonspecific tumor marker, indicating that further prospective randomized controlled trials are needed to validate our findings.

## Conclusion

Our study suggests that SII is a simple and useful prognostic factor for predicting long-term outcomes for resected breast cancer patients, and a high SII suggests poor prognosis.

## Data Availability Statement

The original contributions presented in the study are included in the article/supplementary material. Further inquiries can be directed to the corresponding author.

## Ethics Statement

The present study was approved by the Research Ethics Committee of West China Hospital of Sichuan University, and a written informed consent was obtained from each participant in accordance with the policies of the committee.

## Author Contributions

Data curation: WL, GM, and YD. Investigation: GM and QW. Methodology: YD, WL, and GM. Project administration: QW. Resources: QW. Software: ZL and FC. Visualization: YD. Writing – original draft: WL. Writing – review & editing: QW. All authors contributed to the article and approved the submitted version.

## Conflict of Interest

The authors declare that the research was conducted in the absence of any commercial or financial relationships that could be construed as a potential conflict of interest.

## Publisher’s Note

All claims expressed in this article are solely those of the authors and do not necessarily represent those of their affiliated organizations, or those of the publisher, the editors and the reviewers. Any product that may be evaluated in this article, or claim that may be made by its manufacturer, is not guaranteed or endorsed by the publisher.
